# *eLife* and early career researchers

**DOI:** 10.7554/eLife.01633

**Published:** 2013-10-22

**Authors:** Randy Schekman, Fiona M Watt, Detlef Weigel

**Keywords:** research assessment, careers in science, postdoc, grad school, scientific publishing, science policy

## Abstract

There are many reasons for submitting your best work to *eLife*, especially if you are an early career researcher.

Science students and active researchers are evaluated and assessed many times during their career as they progress from high school to university to graduate school to one or more postdoc positions to eventually—most of us hope—a secure job. As high school students, we were judged by exam results, grade-point averages or standardized test scores. Later, from graduate school onwards, we are mostly evaluated according to the quality of our research. Performing well in such evaluations is essential for anyone who wants to advance in a research environment, so it is crucial that these assessments are performed in a way that is fair and transparent.

The system of evaluation and assessment places substantial pressure on early career researchers, especially when looking for a fellowship or a tenure-track position in a university or research institute. It is not uncommon for job or fellowship announcements to attract hundreds of applications. Many scientists, especially those in the early stages of their career, believe that their chances of succeeding (or even getting an interview) depend primarily on the impact factors of the journals in which they have published. This is more true in some institutions and countries than in others (Ching, 2013). Although more and more funders and institutions are emphasizing that the intrinsic quality of the research is what really matters (DORA, 2013; Schekman and Patterson, 2013; Schmid, 2013), too many scientists still have an unhealthy obsession with getting published in the rarefied world of ‘top-tier’ journals that are characterized by review processes and acceptance policies that often appear opaque and capricious.

And the pressure does not go away after you have landed a tenure-track position, because the next challenges are to secure grant money and to convince your new colleagues that you deserve tenure. (Plus you have to show that you are a good colleague). Again, despite what senior investigators tell them and what the grant-awarding agencies say, many pre-tenure researchers believe that the number of papers in top-tier journals is the key to professional success and happiness. It is worth repeating here that the journal impact factor was never intended to be a measure of the quality of individual research papers: it was designed as a tool for comparing journals (and even then it has certain limitations), and scientists themselves are largely to blame for allowing it to influence decisions about hiring and promotion to the extent that it does (Curry, 2012).

At *eLife*, we recognize these pressures and have introduced a number of measures for the benefit of our colleagues who are in the early stages of their careers. First, we encourage corresponding authors who do not have tenure to mention this in their cover letter. The Senior Editor who handles the manuscript will take this into account when deciding whether or not it merits in-depth peer review by a Reviewing Editor and one or more external referees (Schekman et al, 2013a): as a result, a higher-than-average percentage of manuscripts from early career authors receive in-depth peer review. However, this does not mean that manuscripts from early career authors are more likely to be accepted than those from more established investigators. Rather, it means that early career authors are more likely to receive (and benefit from) the sort of considered, in-depth feedback from referees that will help them to improve the manuscript and increase its chances of publication in *eLife* (or a different journal). And if the Senior Editor decides that a manuscript from an early career researcher should not be sent for in-depth peer review, the authors will in general receive more than just a standard rejection letter.

Second, we recognize that graduate students and postdocs require letters of recommendation when they are applying for jobs and fellowships. Therefore, the Senior Editors of *eLife* have agreed to write a letter of recommendation on behalf of the first author in support of job or fellowship applications, and many authors have requested and received such letters.

The Senior Editors of *eLife* have agreed to write a letter of recommendation on behalf of the first author in support of job or fellowship applications.

In a new effort, we are identifying a small number of particularly outstanding *eLife* Research Articles by early career researchers and inviting the first or corresponding author on each article to give a presentation at a meeting organized by one of the three agencies that sponsor the journal (the Howard Hughes Medical Institute, the Max Planck Society and the Wellcome Trust). Since most *eLife* authors are not funded by these agencies, this initiative gives these early career researchers a valuable opportunity to publicize and discuss their work with audiences that will include many leading researchers in their field. The names of the first four authors to be invited to give such presentations have just been announced (Table 1).

**Table 1.** The first four *eLife*-sponsored presentations by early career researchers

**Rosanna A Alegado** University of California, Berkeley, United States; Present address: University of Hawai`i at Mãnoa	Alegado RA, Brown LW, Cao S, Dermenjian RK, Zuzow R, Fairclough SR, Clardy J, King N. 2012. A bacterial sulfonolipid triggers multicellular development in the closest living relatives of animals. *eLife* **1**:e00013. doi: 10.7554/eLife.00013.
**Jesse D Bloom** Fred Hutchinson Cancer Research Center, Seattle, United States	Gong LI, Suchard MA, Bloom JD. 2013. Stability-mediated epistasis constrains the evolution of an influenza protein. *eLife* **2**:e00631. doi: 10.7554/eLife.00631.
**Israel S Fernandez** Medical Research Council Laboratory of Molecular Biology, Cambridge, United Kingdom	Bai X, Fernandez I, McMullan G, Scheres SHW. 2013. Ribosome structures to near-atomic resolution from thirty thousand cryo-EM particles. *eLife* **2**:e00461. doi: 10.7554/eLife.00461.
**Wenhui Li** National Institute of Biological Sciences, Beijing, China	Yan H, Zhong G, Xu G, He W, Jing Z, Gao Z, Huang Y, Qi Y, Peng B, Wang H, Fu L, Song M, Chen P, Gao W, Ren B, Sun Y, Cai T, Feng X, Sui J, Li W. 2012. Sodium taurocholate cotransporting polypeptide is a functional receptor for human hepatitis B and D virus. *eLife* **1**:e00049. doi: 10.7554/eLife.00049.

The editorial leadership of *eLife* has identified four particularly outstanding Research Articles that were published in the journal before the end of May 2013, and one early career author from each article has been invited to give a presentation at a meeting organized by one of the agencies that sponsor *eLife*. Another four authors will be invited to give presentations early next year.

**Figure 1:**
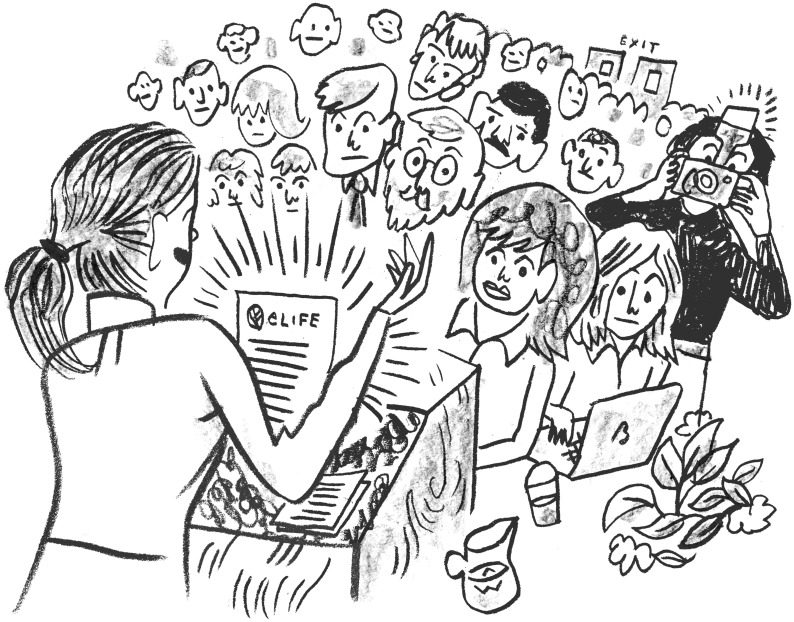
ILLUSTRATION: RIPE.COM

Obviously, we welcome submissions from researchers of all vintages, and we have introduced a number of innovations to improve scientific publishing for the benefit of all authors, such as our innovative approach to peer review, our policy of accepting all manuscripts that meet our (admittedly high) scientific criteria, our commitment to open access, our policy of making the most of digital media by not restricting the number of words, figures or references in a Research Article, our ability to integrate data and video into articles, our sharing of referee reports for rejected articles with a number of other journals, our commitment to extending the reach and impact of articles through plain-language summaries, Insight articles and podcasts, and our progressive media policy, which allows authors to share their results with others ahead of publication if they wish (Schekman et al., 2013b).

We also promise that the initial decision on submissions will be quick—the average is presently 3 days—and that manuscripts will not be subjected to needless cycles of revision and re-review before they are eventually accepted or rejected. Those near the start of their career may have time on their side, but when you have an exciting story to tell, and the competition on the career ladder is intense, the last thing you can afford to happen is for your work to languish in a seemingly endless editorial process.
